# Tuning the Gel Network Structure and Rheology of Acid-Induced Casein Gels via Thiol Blocking

**DOI:** 10.3390/ijms26136206

**Published:** 2025-06-27

**Authors:** Thomas Pütz, Ronald Gebhardt

**Affiliations:** Chair of Soft Matter Process Engineering (AVT.SMP), RWTH Aachen University, 52074 Aachen, Germany; thomas.puetz@avt.rwth-aachen.de

**Keywords:** milk proteins, micellar casein powder, gels, confocal fluorescence microscopy, mechanical characterization

## Abstract

This study systematically investigates how thiol–disulfide interactions influence the structure and mechanical properties of casein gels. Acid gels were prepared from suspensions of micellar casein (MC) powder that were heat-treated at 70 °C. Thiol groups were variably blocked with N-ethylmaleimide (NEM). The gels were characterized using stress–strain measurements, rheological analyses, and confocal microscopy. The stress–strain curves exhibited a biphasic behavior, with an initial linear elastic phase followed by a linear plastic region and a nonlinear failure zone. Compared to control samples, the addition of 100 mM NEM reduced the gel strength by 50%, while G′ and G″ increased by around 100%, unexpectedly. NEM-treated gels consist of uniformly sized building blocks coated with a whey protein layer. Strong physical interactions and dense packing enhance viscoelasticity under short deformations but reduce the compressive strength during prolonged loading. In contrast, control samples without NEM demonstrate weak viscoelasticity and increased compressive strength. The former is attributed to a broader particle size distribution from lower acid stability in the untreated gels, while the particularly high compressive strength of heat-treated gels additionally results from disulfide cross-links. The results show that thiol blocking and heating enable the targeted formation of acid casein gels with high shear stability but a low compressive strength.

## 1. Introduction

Casein, the major milk protein in bovine milk, and whey proteins are sustainable, biobased materials for gel systems. These milk-based protein systems are widely used in food and biotechnology applications due to their high functionality, storage stability, and versatility, such as in beverages, fermented dairy products, and protein-enriched formulations. They also have potential as drug carriers in tissue engineering, wound healing, and diagnostics, particularly as biosensors, offering mechanical strength, stability, biocompatibility, and, where relevant, electrical conductivity [1,2]. Their structured gel networks are the key to tailoring the texture and rheology, with structural modifications crucially affecting the swelling of micellar casein-based fibers and microparticles in applications like encapsulation or tissue scaffolds [3,4,5]. While the role of disulfide bonds in milk protein gels has been extensively studied, the specific impact of thiol blocking in casein gel systems obtained from spray-dried micellar casein (MC) powder—especially regarding their behaviors under shear and compression—remains poorly understood.

The acid-induced gelation of milk proteins is a complex process influenced by heat, pH, protein composition, and both covalent and non-covalent interactions. Particularly, the interactions between casein micelles and thermally denatured whey proteins, such as β-lactoglobulin (β-Lg), largely determine the gel structure and strength. These interactions occur through thiol–disulfide exchange reactions, forming stable, three-dimensional networks [6,7,8].

In bovine milk, about 95% of casein is found as unstructured aggregates called casein micelles. These highly hydrated micelles consist of α_S1_-, α_S2_-, β-, and κ-casein, as well as colloidal calcium phosphate. They vary in diameter size between 80 and 400 nm [9]. In their native state, κ-casein forms a stabilizing “hairy” surface layer that protects the micelles [10,11]. If this stabilizing effect is lost due to acidification or rennet enzymes, the micelles precipitate and aggregate, forming submicron-sized aggregates that, once a critical size is reached, form a coherent gel, as demonstrated by single-particle tracking via fluorescence microscopy [12]. The gel structure links particles like a string of pearls separated by water-filled cavities.

**Figure 1:**
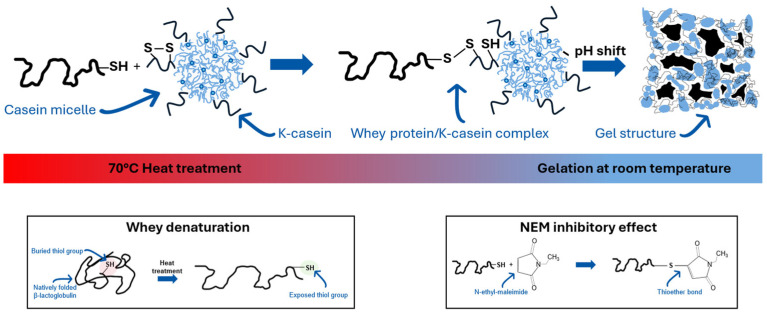
Thiol exchange reaction after temperature activation and NEM’s effect on milk proteins.

Before acidification, milk proteins are often heated above 70 °C, causing whey proteins to denature and interact with casein micelles, particularly through hydrophobic interactions and covalent disulfide bonds involving κ-casein [13,14]. High-temperature treatments above 70 °C lead to high-molecular-weight complexes between denatured whey proteins and κ-casein, which remain partly associated with casein micelles [15]. Subsequently, acidification occurs, causing caseins to precipitate and form a gel matrix. Disulfide bridges and free thiol groups are crucial in this process, determining the gel strength [16,17]. β-Lg is the main source of thiol groups in milk. Its structure consists of disulfide bridges and a free thiol group inside the structure that becomes exposed after thermal unfolding, making it reactive [18,19,20]. Figure 1 shows the interaction between denatured β-Lg and casein micelles. NEM blocks this process by inhibiting the thiol groups of β-Lg, preventing further cross-linking.

However, the gelation process in suspensions of an MC powder must be examined in detail due to its structural particularities, making it crucial to understand the underlying mechanisms that govern this unique behavior. MC powder contains 84% total protein, of which 78.4 ± 0.5% is casein and 5.6 ± 0.6% is whey protein, with 34.6 ± 0.8% in its native form [21,22]. Unlike fresh milk, milk protein concentrates (MPC) are depleted in lactose and serum minerals due to ultrafiltration [23]. MPCs also differ from fresh milk in having reduced urea levels, calcium chelators, and varying calcium ion activities, which lead to different heat stability behaviors [13,24]. MC powder, however, combines microfiltration and diafiltration, allowing some whey proteins, lactose, and serum minerals to pass through. The casein/whey ratio in MC powder is 93:7, which is higher than in fresh milk or MPCs [13,21]. Unlike traditional milk powders, MC powder has recently gained commercial relevance due to its heat stability, making it suitable for sterilized food products without affecting the protein structure [25]. During MC powder manufacturing, whey proteins are already denatured and partially associated with casein micelles, which influences the gelation process [21,26].

These structural features affect how gels form under thermal or chemical conditions, especially the interaction between covalent and physical cross-linking within the network. A key element of these networks are WP/κ-casein complexes that form during heat treatment and exist as micelle-bound or soluble aggregates [20,27,28]. Studies show that bound denatured whey proteins, which covalently bind to casein micelles, significantly increase the storage modulus (G′) of acid gels [28]. In contrast, soluble denatured whey proteins that do not interact with casein lead to weaker gels. However, these gels have a limited gel strength, prompting research on methods to adjust the gel strength [13,14]. Inhibitors like NEM can reduce the gel strength and elasticity, as covalent crosslinks between β-Lg and casein decrease with increasing inhibitor concentration [17,29].

This study investigates gelation mechanisms in systems consisting of MC powder with partially associated whey proteins. MC powder is an increasingly relevant raw material in the food industry due to its unique properties, such as heat stability and water binding. Its market significance is also growing rapidly [30]. However, its gelation properties remain under-explored. While previous studies have demonstrated the impacts of heat treatment and the protein concentration on the microstructure and rheology of acid gels derived from milk protein concentrates [31,32], the significance of covalent bonding, particularly disulfide interactions, remains unclear. This approach addresses a gap in the existing research by establishing a link between the molecular-level chemistry of protein networks—specifically, thiol-based interactions—and the mechanical gel behavior of heat-treated MC systems. These results may contribute to a better understanding and more targeted control of the mechanical properties of acid casein gels derived from MC powder. The inherent whey protein content of MC powder enables disulfide bond formation during heat treatment, which allows for the precise modulation of the gel strength and viscoelastic behavior for use in the food and biomedical industries.

## 2. Results

Differently treated samples were prepared from suspensions of MC powder to investigate the influences of heat and thiol blocking on disulfide bridge formation. Untreated MC served as a control and was left at room temperature. Heat-treated MC was heated to 70 °C for 11 min to induce thermally induced structural changes and possible protein cross-linking. In the other two samples, NEM was added prior to heat treatment to irreversibly block free thiol groups.

**Figure 2:**
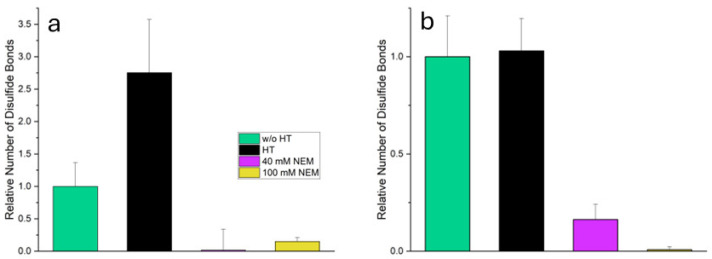
Relative numbers of reducible disulfide bonds in suspensions of micellar casein (MC) after different treatments, as determined with Ellman’s reagent. (**a**) Total system (whey protein + MC), (**b**) supernatant after centrifugation (mainly whey protein).

The disulfide bridges were determined using Ellman’s reagent (DTNB) in combination with a reducing agent. The difference in absorbance with and without reducing agent was used as a measure of the number of reducible disulfide bridges. Figure 2 illustrates how different treatments influence disulfide bridge formation in the total system and the supernatant compared with untreated MC.

In the total system, heat-treated MC shows a significant increase (*p* ≤ 0.05) in the number of disulfide bridges (2.7 times compared to untreated MC) suggesting pronounced heat-induced cross-linking. However, in the samples with added NEM (heat-treated MC + 40 mM NEM and heat-treated MC + 100 mM NEM) the measured number of reducible disulfide bridges is almost zero. This confirms that alkylation by NEM has successfully blocked the thiol groups, thereby preventing their detection by Ellman’s reagent.

In the supernatant, which contains mainly soluble whey proteins, the number of disulfide bridges in heat-treated MC remains almost unchanged compared to untreated MC (*p* ≥ 0.05). This does not indicate an absence of disulfide formation; rather, it reflects the sedimentation of aggregated, unfolded proteins during centrifugation. As a result, they are no longer present in the supernatant and are therefore not detected. These denatured whey proteins expose reactive thiol groups, enabling thiol–disulfide exchange with other whey proteins or casein micelles. This contributes to the increased disulfide bond content observed in the total system. In heat-treated MC + 40 mM NEM, a reduced but still significant proportion of disulfide bridges is detected (0.2-fold), indicating incomplete alkylation. This may be due to the limited accessibility of certain thiol or disulfide groups, which are likely to be embedded within tightly folded whey proteins. The tertiary structure of these proteins may shield reactive groups more effectively than the open structure of casein micelles. In heat-treated MC + 100 mM NEM, the value is almost zero, indicating an almost complete blocking of the thiol groups by the higher NEM concentration. Building on these findings regarding disulfide bridge formation, the subsequent subchapters will examine the microstructure of the gels in detail, as well as their mechanical properties when subjected to shear and compressive stress.

### 2.1. Microstructure of the Gels

**Figure 3:**
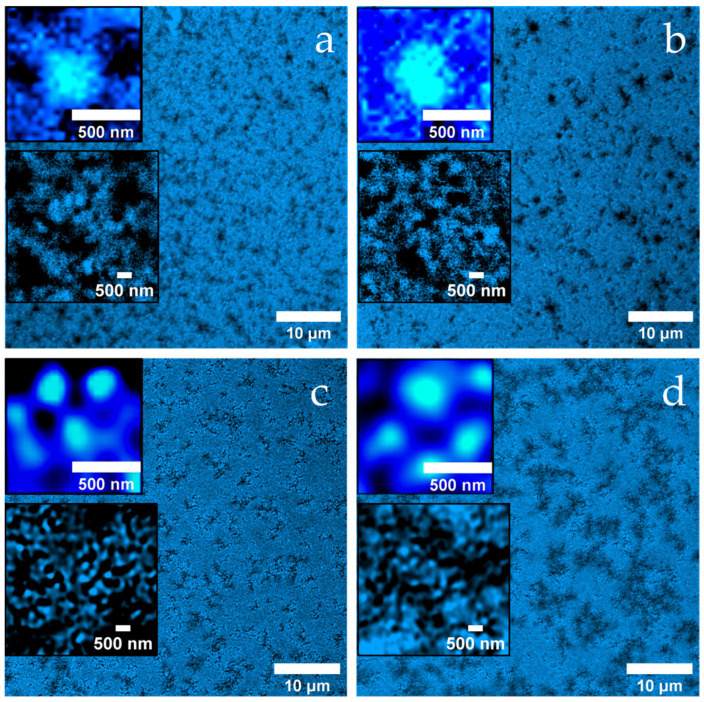
Confocal fluorescence micrographs of casein acid gels from casein micelles (**a**) without temperature treatment, (**b**) with temperature treatment, (**c**) with temperature treatment and 40 mM added NEM, and (**d**) with temperature treatment and 100 mM added NEM. In each image, insets (1 µm × 1 µm and 5 µm × 5 µm) highlight the typical microstructure at higher magnification, allowing a detailed visualization of the aggregate morphology and substructure.

Figure 3a reveals a loosely connected, porous gel network of the untreated MC, which consists of hierarchically structured aggregates. The dark background indicates the absence of denatured whey proteins in the continuous phase.

After heat treatment (Figure 3b), the gel has a much more compact appearance, with shorter, partially widened channels, some of which look almost like cavities. Again, the aggregates consist of approximately 400 nm units with substructure (see insets), but the background is now fluorescent, indicating denatured whey proteins that may not have been fully incorporated into the network.

In Figure 3c (heat treatment + 40 mM NEM) the network appears finer meshed with uniform channels of an approximately 500 nm width. The aggregates are smoother and smaller and are surrounded by a thin fluorescent shell, which is likely due to the presence of adsorbed denatured whey proteins.

Figure 3d (heat treatment + 100 mM NEM) shows a similar submicroscopic structure (see inset), but the channels between the aggregates have widened significantly to around 2 µm. This widening is likely due to the formation of more compact, densely packed aggregate units that create larger interstitial spaces, despite an overall tighter local structure. Thus, the strong inhibition of disulfide bridge formation at high NEM concentrations appears to promote the formation of larger, smoother aggregates and a more organized yet more compartmentalized network.

### 2.2. Gel Firmness During Shearing

**Figure 4:**
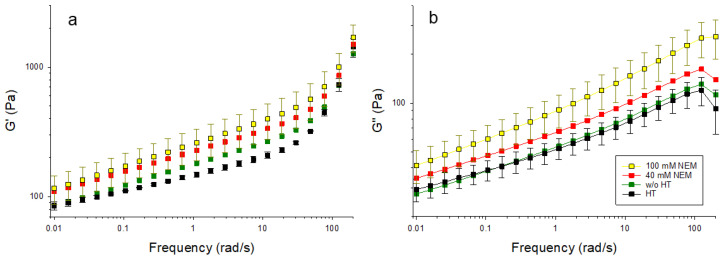
Changes in viscoelastic behavior due to addition of NEM before gel formation. (**a**) Storage modulus G′ for elastic behavior and (**b**) loss modulus G″ for viscous behavior.

Acid gels containing differently pre-treated micellar casein were subjected to oscillatory shearing to investigate the effect of the disulfide network on the viscoelastic behavior. The material functions storage modulus (G′) and loss modulus (G″) are presented in Figure 4 over a wide frequency range. G′ reflects the elastic component of the material, indicating its ability to store deformation energy, while G″ represents the viscous component, corresponding to energy dissipation. Typically, a higher G′ denotes a more solid-like behavior, whereas an increased G″ is associated with a more fluid-like response. These rheological parameters are commonly used to evaluate the integrity of the gel network and its resistance to deformation and shear. With increasing NEM concentrations, both G′ and G″ increase over the whole frequency range without the shape of the curve changing significantly. The latter indicates that the mechanism of how the gels respond to shear is little changed by NEM. However, the increases in the absolute values of G′ and G″ suggest increases in gel strength and internal friction. These may be due to the altered microstructure rather than primarily being driven by disulfide cross-linking, which is greatly reduced in the presence of NEM. Two frequency ranges with different behaviors can be identified for G′ (Figure 4a). In the first range, up to about 20 rad/s, there is a linear increase in the double logarithmic plot for all gel samples. Over the entire frequency range, G′ is highest for the 100 mM NEM gel, followed by the 40 mM NEM gel, and the untreated gel, and the lowest values are observed for the heat-treated gel without NEM. At higher frequencies (>20 rad/s to 200 rad/s), G′ continues to increase linearly for all samples, but at a significantly higher rate, with the increase being more pronounced for the gels without NEM compared to those with NEM.

G″ (Figure 4b) also increases linearly in the double-log plot for all samples up to 100 rad/s. The untreated gel and the heat-treated gel without NEM show almost identical behaviors, while the gels with NEM again (40 mM and 100 mM) show higher G″ values. The highest G″ values are observed in the gel with 100 mM NEM.

In combination with the microscopy data, these findings suggest that thiol blocking promotes the formation of more compact aggregate structures with tighter physical contacts, thereby increasing shear resistance.

### 2.3. Gel Firmness in the Compression Test

**Figure 5:**
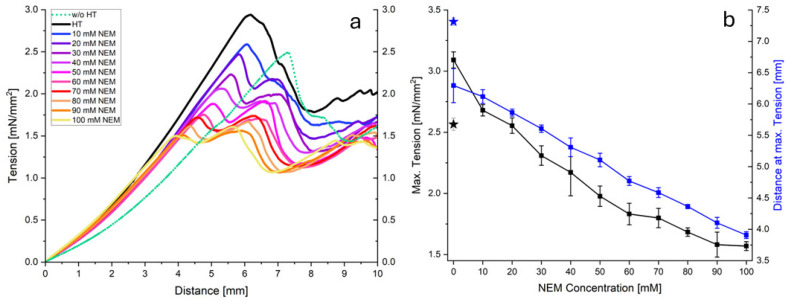
Pressure versus penetration depth measurements (**a**) and material parameters obtained (**b**) for gels of temperature-pretreated casein micelles mixed with different concentrations of NEM compared to the reference without the temperature pre-treatment shown as stars.

In addition to the shear effect, the behavior of the gels under a compressive load was investigated. Figure 5a shows the stress–strain curves obtained for acid gels prepared from micellar casein that was pretreated at 70 °C with different concentrations of NEM. Generally, all curves exhibit a linear, two-phase, progressive course to a maximum value (Figure 5a). As the NEM concentration increases, it is clear that both the increase in the second phase and the maximum value decrease. Thereafter, the stress decreases due to irreversible gel damage, which was not considered in the subsequent analysis. Figure 5b plots the maximum stress (peak maximum) and the distance at maximum tension as a function of the NEM concentration. Both the maximum stress and distance decrease almost linearly with increasing NEM concentrations, indicating a reduced gel strength under large compressive deformations. This is likely due to fewer disulfide cross-links stabilizing the network at higher strains, despite the more cohesive gel structure observed under small shear deformations. Although NEM-treated gels have denser, more compact aggregate structures, confocal images also revealed enlarged inter-aggregate channels at higher NEM concentrations. Under a large compressive strain, these voids compact more rapidly. Without covalent reinforcement, this results in early mechanical collapse.

To analyze the elastic–plastic gel response up to the maximum stress, we applied a model that links two linear force-displacement regions characterized by slopes k_1_ and k_2_ via a transition function (TF):

(1) $$F\left(d\right)=k_{1}\cdot d+\left(k_{2}-k_{1}\right)\cdot \left(d\right)\cdot TF$$

with

(2) $$TF=\frac{1}{1+e^{-k_{T}\left(d-d^{*}\right)}}$$

**Figure 6:**
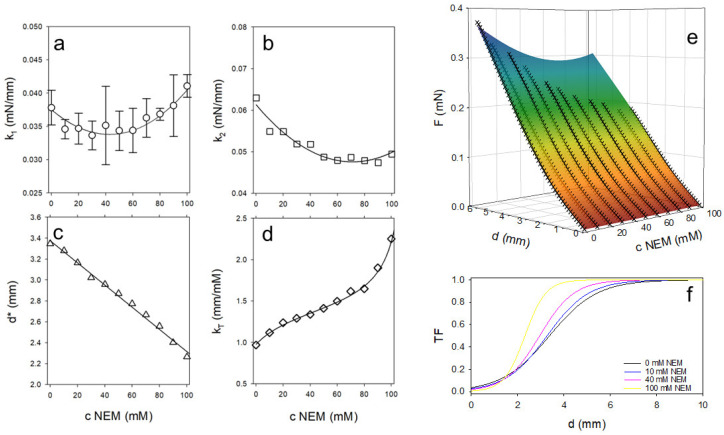
Values of the parameters of model 1 (**a**–**d**) obtained by fitting Equation (1) to the individual measured curves in Figure 5a. The simultaneous fit is shown as a hypersurface of all force–displacement curves as a function of the NEM concentration (**e**) using Equations (3)–(5), with simulated curves also shown as lines in a-d. Calculated transition functions for selected NEM concentrations are shown (**f**).

If d << d*, the denominator of TF becomes very large and TF → 0. As a result, the first summand of Equation (1) dominates the model curve. On the other hand, if d* >> d, the exponential term tends to 0 and the second summand describes the further course of the measurement curve. Equation (1) was fitted to the original data (force instead of pressure) using a non-linear fit. The values of the fit parameters are plotted as symbols in Figure 6a–d. As shown in Figure 6a, the values for k_1_ change in a parabolic fashion with the NEM concentration. Up to approximately 40 mM NEM, the slope of the force–displacement curves initially decreases and then increases as the concentration of thiol blocker is further increased. In contrast, the slope of the second part of the curve, k_2_, decreases degressively with increasing NEM concentrations. The value d* indicates the depth of penetration into the gel at which TF = ½ and thus the transition between the two linear sections occurs. Figure 6c shows that d* decreases linearly with increasing NEM concentration. The steepness of the transition, described by k_T_, initially increases to a plateau value at intermediate NEM concentrations and then increases steeply again, as shown in Figure 6d. The symmetry of the values for k_1_ and k_T_ around a central region indicates two different gel structures induced by the addition of NEM. In order to characterize the transition region via a characteristic NEM concentration c*, Equation (1) was extended to include concentration-dependent functional relationships for the fit parameters, where simple parabolas have been chosen for k_1_ and k_2_ via

(3) $$k_{1/2}\left(c\right)=z_{0,1/2}+a_{1/2}\cdot {\left(c-c_{1/2}^{*}\right)}^{2}$$

a linear function for d* via

(4) $$d^{*}\left(c\right)=z_{0,d}+a_{d}\cdot c$$

and a point symmetric function for k_T_ via

(5) $$k_{T}\left(c\right)=\alpha +\beta \cdot tan\left(\gamma \cdot \left(c-c_{1}^{*}\right)\right)$$

The parameter fits (Table 1) reveal a characteristic NEM concentration of c* = 42.7 mM, which marks a structural transition in the gel’s behavior. The model fits the data well, with an R^2^ value of 0.998 and a standard error of estimate of 0.0033, validating the applied approach. As the NEM concentration increases, the transition between the elastic and plastic regions becomes steeper and occurs at shallower penetration depths (see Figure 6f), which is consistent with the formation of more compact and uniform gel structures that can be observed microscopically.

The results for disulfide bonding, the microstructure, and mechanical behavior are discussed together in the following section to show how they are linked and what they mean in practice.

## 3. Discussion

This study investigates how heat treatment and thiol blocking with NEM influence disulfide bond formation in micellar casein suspensions, and how this impacts the mechanical properties of acid gels. It is well established that disulfide bonds enhance gel stability and texture. Xia et al. reported that adding plant proteins such as pea protein reduces the effectiveness of these bonds, resulting in poorer rheological properties, including decreased elasticity, cohesiveness, and chewiness [33]. Similarly, Alting et al. demonstrated that the hardness of whey protein gels is primarily dependent on disulfide cross-linking rather than the aggregate size; blocking thiol groups was found to significantly weaken the gels [34]. In contrast, our results reveal a concentration-dependent effect of thiol blocking by NEM, where gels show increased shear stability but reduced compressive strength. Our findings reveal a complex interplay between molecular interactions and mechanical properties. This relationship was not fully captured in previous studies. It is also relevant in this context that the micellar casein powder (MC88) used in our study has a high protein content. Such powders have been shown to retain κ-casein in the micellar phase upon heating and resist dissociation, as described by Meletharayil et al. [32].

**Figure 7:**
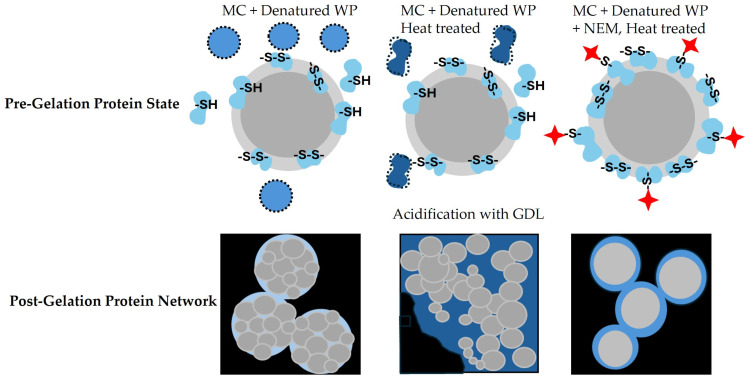
Schematic representation of the protein states before and after the acid-induced gelation of micellar casein-based systems. The upper row illustrates the structural organization of micellar casein (MC), denatured whey proteins, and their interactions prior to gelation under different treatments. NEM blocks free thiol groups as indicated by red stars. The lower row shows the resulting gel microstructures after acidification.

The gel networks of the untreated reference sample and the heat-treated micellar casein sample without NEM consisted of differently sized particles. These formed as a result of acid-induced alterations in micellar structure [35]. The gel obtained from heat-treated micellar casein without NEM showed a more compact microstructure compared to the untreated reference. In particular, background fluorescence was observed, indicating the presence of unbound denatured whey protein aggregates (Figure 7, bottom row). Aggregates are highlighted in the schematic representation of the initial state, where the denatured whey protein aggregates are shown as freely dispersed or partially associated with the micellar surfaces (Figure 7, top row). These aggregates are likely to intercalate between the casein micelles and weaken the cohesion of the network at the molecular level, resulting in a slightly less cohesive gel. Accordingly, this sample exhibited the lowest G′ and G″ values across all conditions tested, indicating reduced shear stability and reduced elastic and viscous resistance. This finding is consistent with the observations of Lucey et al. [28], who reported that denatured whey proteins only contribute to an increase in G′ when they associate effectively with casein micelles during heat treatment. However, if the proteins remain unbound or loosely associated, as suggested by our fluorescence microscopy data, they may interfere with network formation and reduce gel strength. This observation of reduced G′ seems to contradict statements by Asaduzzaman et al., who noted that heating milk at natural pH results in curds with higher G′ and lower tan δ due to balanced contributions from casein and whey protein complexes [36]. Similarly, Wu et al. found that gel elasticity and G′ values increased with higher heat treatment temperatures [37]. They found that preheating skim milk to 147 °C before gelling produced gels with greater elasticity than those preheated to 142 °C. This effect is attributed to the formation of more disulfide bonds, which are essential for firm, water-binding protein gels [37]. Our observations can, however, be explained by Nikolai et al., who noted that whey proteins can only enhance the gel strength when sufficiently heated (>70 °C). At temperatures near this threshold, as in our study, whey proteins may interfere with gel formation by chelating calcium, thus disrupting the cross-linking capacity of casein micelles [31]. This is supported by the diffuse background fluorescence observed, which indicates the presence of calcium-binding aggregates that reduce the availability of casein micelles for gel formation. This likely contributes to the lower G′ values, despite the disulfide-mediated structural enhancement. These discrepancies highlight the importance of the extent of heat pretreatment as a critical influencing factor, which may limit the generalizability of previous findings to the conditions applied in our study.

However, in compression tests gels derived from heat-treated MC suspensions without NEM exhibited the highest fracture stress, suggesting mechanical reinforcement likely mediated by disulfide cross-links between denatured whey proteins and casein micelles, despite its limited resistance to shear deformation. These covalent bonds appear to be more important under conditions of plastic deformation, where they increase the load-bearing capacity of the gel network.

In contrast, gels containing NEM (40 and 100 mM) showed smaller, smoother aggregates with no internal substructure and peripheral fluorescence, suggesting surface-bound hydrophobically associated whey proteins. The initial state of these gels (Figure 7, top row) shows the presence of thiol-blocked denatured whey proteins that bind to the surface of the casein micelles, forming a protective layer around them. This protective layer prevents the 250 nm casein particles/micelles from disintegrating during acidification, which was the case in the samples without NEM. This aligns with the observation by Lucey et al., who found that small amounts of denatured whey proteins associated with casein micelles during low-heat processing resulted in stronger gels with higher G′ values [28]. These gels with NEM exhibited the highest G′ and G″ values across the frequency range, indicating increased shear stability and a denser, physically stabilized network. G′ remained greater than G″ across the entire frequency range, confirming the predominantly elastic nature of these gels. Despite thiol blocking, these increases in the viscoelastic moduli can be explained by the aggregate structure becoming more uniform and compact. Systems with a narrow particle size distribution are known to exhibit enhanced viscoelastic properties at the same volume fraction due to more effective interparticle interactions and network connectivity [38]. This interpretation is supported by our microstructural observations revealing smaller, smoother, and more homogeneous aggregates in NEM-treated gels.

The normal force tests show a two-phase response with two linear force increases: the first corresponding to elastic behavior and the second reflecting plastic deformation due to polymer chain slippage. The maximum force decreases almost linearly with increasing NEM concentration, while the penetration depth at which this is achieved also decreases continuously. This shows that the plastic deformability of the material is reduced with increasing NEM concentrations and the gel becomes less resistant. These findings are consistent with those of Vasbinder et al., who demonstrated that the presence of whey protein aggregates significantly increases gel hardness, whereas NEM-induced thiol blockade reduces gel hardness by preventing disulfide bond formation between protein components [29]. Our observation of a high gel hardness but relatively low storage modulus in gels containing whey protein aggregates supports the findings of Lucey et al., although Vasbinder et al. questioned this correlation based on discrepancies between force–distance slopes and modulus data [28,29]. This duality between gel hardness and storage modulus is also reflected in our own experiments, in which an increase in the NEM concentration changes the mechanical properties of the gel, particularly with regard to plastic deformation.

The initial elastic increase in the force–displacement curve shows a parabolic course as a function of NEM concentration, as illustrated in Figure 6a: it decreases up to average NEM concentrations of c* = 42 mM and increases again at higher concentrations. This can be explained by the disappearance of large rigid aggregates, followed by an increasing stiffness of the remaining matrix. In contrast, the second, plastic increase decreases continuously, indicating a higher polymer chain mobility with increasing NEM concentrations. Due to the reduced cross-linking via disulfide bridges, the chains can slide more easily, which means that the gel offers less resistance to plastic deformation. This increased mobility also explains the lower maximum force and the earlier occurrence of this force at lower penetration depths as the gel becomes more flexible overall.

The initial broad transition curve between the elastic and plastic regions becomes progressively sharper with increasing NEM concentrations and takes on an increasingly stepped shape. This indicates an abrupt change in resistance to plastic deformation. The increasing homogeneity of the mechanical properties of the gel is supported by the changes in the microstructure discussed above.

Taken together, these results demonstrate that NEM modulates gel properties by altering the microstructure and cross-linking capacity. While NEM increases shear resistance by promoting physically stabilized protein structures with a more uniform particle size distribution, it reduces covalent cross-linking, thereby decreasing the compressive strength. This dual effect highlights the complex interplay between molecular interactions and macroscopic gel mechanics. Applying a simultaneous fit model to the compression data enables us to capture the NEM- and deformation-dependent mechanical response in a unified manner. This validated model supports predictions beyond the experimental conditions and provides a solid basis for investigating additional factors, such as the influence of the shear rate under pressure. It also facilitates the clear identification of functional relationships between the fit parameters and the NEM concentration.

Altogether, the results demonstrate that thiol blockers and heat treatment can be used to create casein gels derived from MC powder that are highly shear-stable. These gels are therefore suitable for use in the pharmaceutical and food industries for applications such as encapsulation, where they can protect sensitive ingredients and maintain their structure under mechanical stress. However, their lower resistance to compressive forces suggests that further research is needed, particularly under conditions of high deformation or pressure, to improve their robustness for broader industrial use. These insights contribute to a deeper understanding of structure–function relationships in MC gels and support the design of customized food and biomaterial systems.

## 4. Materials and Methods

### 4.1. Chemicals

For the suspensions, micellar casein powder MC88 was used (MILEI GmbH, Leutkirch im Allgäu, Germany). N-Ethylmaleimide (NEM) was obtained from Sigma-Aldrich (Sigma-Aldrich, Saint Louis, MO, USA). Glucono delta-lactone (GDL) was bought from Thermo Fisher (Thermo Fisher, Waltham, MA, USA). Simulated milk ultrafiltrate (SMUF) was prepared according to the composition proposed by Dumpler [39]. All salts for the SMUF solution were of analytical grade. To inhibit bacterial growth, 0.5 g/L sodium azide was added to the mixture.

### 4.2. Preparation of Casein Suspensions

MC88 was added to SMUF to obtain a final casein concentration of 5% *w*/*w*. The mixture was stirred at room temperature for 1 h, at 4 °C for 16 h, and for 1 h at 37 °C to fully hydrate the casein micelles. NEM was added at concentrations from 0–100 mM and the solution was then gently stirred at 30 °C for 24 h to reduce inhomogeneities in the gel, as observed from previous experiments and confocal images. 

### 4.3. Heat Treatment and Gelation

To initiate the denaturation of β-lg, the solution was heated at 70 °C for 11 min and subsequently cooled in ice water for 10 min. A total of 0.5 g GDL/g casein was added to initiate the gelation process, reducing the pH from 6.65 to 4.15. The mixture was stored at room temperature for about 20 h prior to testing.

### 4.4. Mechanical Tests

Prior to gelation, 20 g of the solution was poured into 50 mL glass beakers with a 4.2 cm diameter. Deformation tests were performed with the texture analyzer AGS-X (Shimadzu, Kyoto, Japan). A circular plunger with a 12.7 mm diameter penetrated the gels and a force–distance curve was obtained at a crosshead speed of 0.1 mms^−1^. Gel hardness was expressed as the maximum stress at the maximum peak of the force–distance curve [40]. Elasticity was determined in the range from 0 to 1 mm, where the lines show linear progression and reversibility (see the Supplementary Figure S1). All experiments were at least performed in triplicate.

In addition to the descriptive analysis, we analyzed all force–displacement curves obtained at varying NEM concentrations by non-linear simultaneous fitting of Equations (3)–(5). We implemented these equations and fitted them to the data using SigmaPlot 14.0 (Systat Software Inc., San Jose, CA, USA). This novel modelling approach is introduced and elaborated upon further in the Results section (see Section 2.3 and Figure 6).

### 4.5. Rheology

The storage modulus G′ and loss modulus G″ were determined using a MCR501 Rheometer (Anton Paar, Graz, Austria). Measurements were performed using the plate–plate geometry PP25 with a stainless steel plate (d = 25 mm) and tinplate single-use cups (d = 65 mm), which were filled with 3.5 g of the casein solution prior to gelation. The gap height was set to 1 mm. After the application of the axial force, the edges of the sample were removed using a scalpel. Liquid paraffin oil was applied to the edges to reduce evaporation. Additionally, a solvent trap further minimized moisture evaporation. Then, the sample was allowed to rest for five minutes prior to testing to reduce any tension effects due to application. Frequency sweeps were carried out at a 0.65% constant strain, which was found to be within the linear viscoelastic range determined by an amplitude test. Frequency sweeps were carried out between a range of 0.01 and 200 rad/s. All measurements were performed at room temperature and at least in triplicate. Error bars (±SDs, n = 3) for G′ and G″ are shown for the most relevant samples to illustrate the measurement variability. Although the standard deviations of the remaining groups overlapped across the entire frequency range, consistent increases in the mean values of G′ and G″ were observed with increasing NEM concentrations. As this is a descriptive analysis, no further statistical testing was performed.

### 4.6. Confocal Scanning Laser Microscopy

Imaging was performed using the LSM 980 Airyscan 2 confocal scanning microscope (Carl Zeiss Microscopy GmbH, Jena, Germany). Subsequent to GDL addition, the solution was stained with the fluorescent dye pyranine (Sigma-Aldrich, Saint Louis, MO, USA) by adding 1 mL of a 2 mM pyranine solution prepared in SMUF. A total of 200 µL was then transferred onto glass slides for gelation. Excitation was performed at 340 nm and emission was detected at 512 nm. To ensure comparability across samples, all images were acquired under identical conditions, including protein and fluorophore concentrations, as well as microscope settings.

### 4.7. Determination of Disulfide Bonds

Disulfide bonds in the samples were reduced as follows: 5 µL of the casein solutions was pipetted into a 96-well plate. Two sets of samples were prepared: one group was treated with Ellman’s reagent 5,5′-dithiobis(2-nitrobenzoic acid) (DTNB, Apollo Scientific, Stockport, UK) alone, and the other with DTNB and Tris(2-carboxyethyl)phosphine hydrochloride (TCEP, Apollo Scientific, UK). For the cleavage of disulfide bonds, 5 mM TCEP was added to the samples in the DTNB + TCEP group. The wells were filled with SMUF up to a final volume of 150 µL, followed by an incubation at room temperature for 30 min in a shaking incubator. Following reduction, free thiol groups were quantified using DTNB. Samples were incubated with 1.5 mM DTNB at room temperature for 15 min in a shaking incubator. To isolate the serum phase, the samples were centrifuged at 70,100× *g* and 25 °C for 1 h using an Optima XPN-80 ultracentrifuge (Beckman Coulter, Brea, CA, USA). The absorbance was measured at 412 nm using a BioTek Snyergy HTX microplate reader (Agilent Technologies, Santa Clara, CA, USA), and baseline scattering (800–900 nm) was subtracted from all measurements. All measurements were performed in triplicate. For the relative quantification of disulfide bonds, the difference in optical density was calculated between the control samples (DTNB only) and the samples treated with DTNB + TCEP.

## 5. Conclusions

This study investigated the influence of blocked thiol groups on the structure and mechanical properties of casein-based gels, all subjected to an identical heat treatment. By selectively blocking free thiol groups prior to denaturation, the formation of covalent disulfide bridges between whey proteins and casein was suppressed. Spherical protein aggregates were observed in all samples. However, the thiol-blocked gels exhibited significantly smaller aggregates and smooth surfaces without an internal substructure. This was probably due to reduced hydration and increased physical aggregation.

A particularly striking feature was the complementary mechanical behaviors. Under shear stress, the thiol-blocked gels showed the highest storage (G′) and loss moduli (G″) of all samples tested. Both moduli increased with higher blocker concentrations. However, these networks proved to be pressure-sensitive in compression tests, failing after only minimal deformation. In contrast, the heat-treated sample without NEM showed the highest fracture stress, suggesting that disulfide cross-linking plays a critical role in the compressive strength. This ambivalent behavior suggests the formation of dense, physically stabilized network structures that are highly resistant to shear but less resilient under compressive load due to suppressed covalent cross-linking.

The results suggest that by selectively modifying the cross-linking mechanisms, it is possible to precisely control the mechanical properties of the gels. Products requiring specific properties, such as high spreadability while retaining structural integrity under load, could benefit from cross-linked gel structures, which are obtained through controlled thermal treatment.

In the future, the use of additional modifiers, such as specialized additives or blockers, could further optimize the balance between shear stability and compressive strength. Overall, the deliberate separation of covalent and physical network formation opens up new possibilities for the development of innovative, functional food structures. These findings highlight the potential of micellar casein powder as a tunable protein system. The interplay between thermally induced disulfide bonding and physical aggregation can be exploited to engineer gel networks with customized mechanical responses for targeted food and biomedical applications.

## Figures and Tables

**Table 1:** Parameters of the elastic–plastic transition model (Equations (1)–(5)) with values obtained by non-linear fitting of individual equations (regular font) or by simultaneous fitting of the entire model to all data (bold font).

Parameter	Unit	Value
$z_{0,1}$	mN∙mm^−1^	**3.4** **× 10^−2^**
$z_{0,2}$	mN∙mm^−1^	**4.8** **× 10^−2^**
$z_{0,d}$	mm	3.38
$a_{1}$	mN∙mm^−2^	**2** **× 10^−6^**
$a_{2}$	mN∙mm^−2^	**2.7** **× 10^−6^**
$a_{d}$	mm∙mM^−1^	−1.1 × 10^−2^
$c_{1}^{*}$	mM	42.7
$c_{2}^{*}$	mM	**71.1**
α	mm^−1^	1.37
β	mm^−1^	0.3
γ	mM^−1^	1.37

## Data Availability

The original contributions presented in this study are included in the article/Supplementary Materials. Further inquiries can be directed to the corresponding author.

## Supplementary Materials

The supporting information can be downloaded at https://www.mdpi.com/article/10.3390/ijms26136206/s1.

## Abbreviations

The following abbreviations are used in this manuscript:

DTNB	5,5′-Dithiobis(2-nitrobenzoic acid)
MC	Micellar casein
MPC	Milk protein concentrate
NEM	N-Ethylmaleimide
TCEP	Tris(2-carboxyethyl)phosphine hydrochloride
β-Lg	β-Lactoglobulin
