# Supplementary material for: Tuning the Gel Network Structure and Rheology of Acid-Induced Casein Gels via Thiol Blocking
Source: Int J Mol Sci. 2025 Jun 27;26(13):6206. doi: 10.3390/ijms26136206 (PMC12249982; doi:10.3390/ijms26136206)
Supplement: Supplementary file 1 [file ijms-26-06206-s001.zip › ijms-3651182-supplementary.pdf]

Supporting Information

Tuning the Gel Network Structure and Rheology of Acid-Induced Casein Gels via Thiol Blocking

*Thomas Pütz and Ronald Gebhardt**

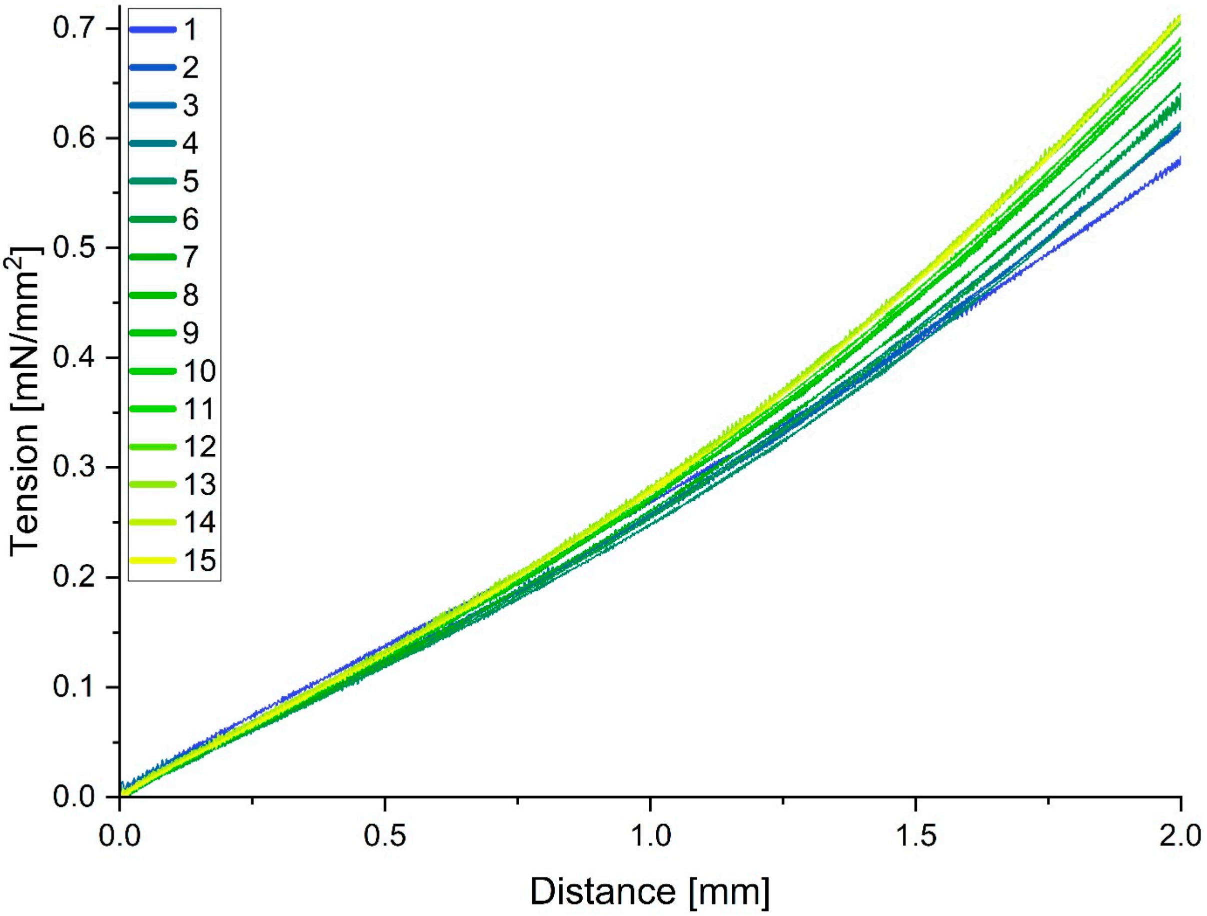

Figure S1. Strain-stress curves of an acid-induced casein gel without heat treatment. Fifteen repeated measurements were performed at identical locations on the same sample with a fixed penetration depth of 2 mm to determine the reversible linear elastic (up to ~1 mm) and the irreversible linear plastic range.
